# Monoclonal antibodies block transmission of genetically diverse *Plasmodium falciparum* strains to mosquitoes

**DOI:** 10.1038/s41541-021-00366-9

**Published:** 2021-08-12

**Authors:** Roos M. de Jong, Lisette Meerstein-Kessel, Dari F. Da, Sandrine Nsango, Joseph D. Challenger, Marga van de Vegte-Bolmer, Geert-Jan van Gemert, Elias Duarte, Noam Teyssier, Robert W. Sauerwein, Thomas S. Churcher, Roch K. Dabire, Isabelle Morlais, Emily Locke, Martijn A. Huynen, Teun Bousema, Matthijs M. Jore

**Affiliations:** 1 Department of Medical Microbiology and Radboud Center for Infectious Diseases, Radboud University Medical Center, Nijmegen, The Netherlands; 2 Center for Molecular and Biomolecular Informatics, Radboud University Medical Center, Nijmegen, The Netherlands; 3 Institut de Recherche en Sciences de la Santé, Direction Régionale, Bobo Dioulasso, Burkina Faso; 4 Malaria Research Laboratory, OCEAC, Yaoundé, Cameroon; 5 Faculty of Medicine and Pharmaceutical Sciences, University of Douala, Douala, Cameroon; 6 Medical Research Council Centre for Global Infections Disease Analysis, Department of Infectious Disease Epidemiology, Imperial College London, London, UK; 7 EPPIcenter Research Program, Division of HIV, ID, and Global Medicine, Department of Medicine, University of California, San Francisco, CA USA; 8 MIVEGEC, Université Montpellier, IRD, CNRS, Montpellier, France; 9 PATH’s Malaria Vaccine Initiative, Washington, DC USA; 10 Present Address: TropIQ Health Sciences, Nijmegen, Netherlands

**Keywords:** Parasitic infection, Vaccines, Microbiology

## Abstract

Malaria parasite transmission to mosquitoes relies on the uptake of sexual stage parasites during a blood meal and subsequent formation of oocysts on the mosquito midgut wall. Transmission-blocking vaccines (TBVs) and monoclonal antibodies (mAbs) target sexual stage antigens to interrupt human-to-mosquito transmission and may form important tools for malaria elimination. Although most epitopes of these antigens are considered highly conserved, little is known about the impact of natural genetic diversity on the functional activity of transmission-blocking antibodies. Here we measured the efficacy of three mAbs against leading TBV candidates (Pfs48/45, Pfs25 and Pfs230) in transmission assays with parasites from naturally infected donors compared to their efficacy against the strain they were raised against (NF54). Transmission-reducing activity (TRA) was measured as reduction in mean oocyst intensity. mAb 45.1 (α-Pfs48/45) and mAb 4B7 (α-Pfs25) reduced transmission of field parasites from almost all donors with IC_80_ values similar to NF54. Sequencing of oocysts that survived high mAb concentrations did not suggest enrichment of escape genotypes. mAb 2A2 (α-Pfs230) only reduced transmission of parasites from a minority of the donors, suggesting that it targets a non-conserved epitope. Using six laboratory-adapted strains, we revealed that mutations in one Pfs230 domain correlate with mAb gamete surface binding and functional TRA. Our findings demonstrate that, despite the conserved nature of sexual stage antigens, minor sequence variation can significantly impact the efficacy of transmission-blocking mAbs. Since mAb 45.1 shows high potency against genetically diverse strains, our findings support its further clinical development and may inform Pfs48/45 vaccine design.

## Introduction

Malaria, caused by the unicellular parasite *Plasmodium* spp., continues to cause high mortality and morbidity worldwide^1^. Current tools, while demonstrating great impact, are considered insufficient to eliminate malaria from most African regions^2^. One tremendous challenge for malaria control and elimination is the efficient spread of malaria to mosquitoes that starts with the uptake of circulating sexual stage parasites, gametocytes, by the mosquito vector during a blood meal on an infected individual. In the mosquito midgut, gametocytes egress from the host red blood cells and develop into gametes. Male gametocytes produce up to eight motile microgametes upon exflagellation and female gametocytes develop into one immotile macrogamete. Zygotes are formed upon fertilization of a macrogamete by a microgamete^3,4^. The zygote develops into a motile ookinete that is able to traverse the midgut wall to establish an oocyst^5^. After differentiation and replication inside the oocyst, parasites are released as sporozoites that migrate to the salivary glands and render the mosquito infectious.

Transmission-blocking vaccines (TBVs) aim to induce antibodies that are taken up by the mosquito vector together with the infectious blood meal containing gametocytes. In the mosquito midgut, these antibodies bind to surface antigens on sexual stage parasites and thereby interfere with sexual development. Three sexual stage antigens are currently under clinical development and are leading TBV candidates: Pfs48/45, Pfs230, and Pfs25. Pfs48/45 and Pfs230 are expressed on the surface of gametes and antibodies targeting these antigens prevent fertilization^6–9^. Antibodies against Pfs25 target zygotes and ookinetes and prevent oocyst formation^6,8,9^. Development of these vaccine candidates has been hampered by challenges with recombinant protein expression and replication of pre-clinical successes. The first versions of Pfs25-based vaccines have been tested in both naive healthy adults and in malaria-exposed individuals^10–13^. Recently, Pfs230-based vaccines have also entered phase I studies (ref. ^14^ and clinicaltrials.gov: NCT02942277), as well as a vaccine targeting Pfs48/45 (clinicaltrials.gov: NCT04862416).

While the development of a highly effective TBV formulation is still challenging, a panel of potent monoclonal antibodies (mAbs) targeting these antigens is readily available. These have been isolated from immunized rodents and block development of cultured parasites in in vitro standard membrane feeding assays (SMFAs)^15^. These mAbs provide insight into protective epitopes and as such may inform vaccine design and development^16^. In addition, passive immunization with mAbs can form an alternative immunization strategy that conveys predictable high-level protection. Fc modifications that extend the serum half-life of immunoglobulin (IgGs)^17^ make it conceivable that efficacious concentrations of mAbs can be sustained for periods that are sufficiently long to support malaria elimination initiatives, contain outbreaks or span seasonal peaks of transmission.

Given the genetic diversity of parasites in endemic settings, cross-strain protection is crucial for the efficacy of both active and passive immunization strategies. Asexual stage antigens in particular are highly polymorphic and vaccines targeting these antigens face challenges to induce cross-strain protection^18^. The general consensus is that sexual stage antigens are well conserved (Supplementary Fig. 1); natural genetic variation may thus have limited impact on TBVs and antibody efficacy. Nevertheless, genetic variation has been observed, especially in Pfs230^19^. Given their increasing prominence in malaria vaccine development, it is both timely and important to assess whether active and passive immunization strategies are likely to encounter challenges due to genetic diversity. Here we compared the efficacy of mAbs targeting Pfs48/45, Pfs230 and Pfs25 in membrane feeding assays against cultured parasite strains and parasites derived from naturally infected gametocyte carriers in Cameroon and Burkina Faso.

## Results

### Three mAbs strongly reduce transmission of reference strain NF54

**Fig. 1:**
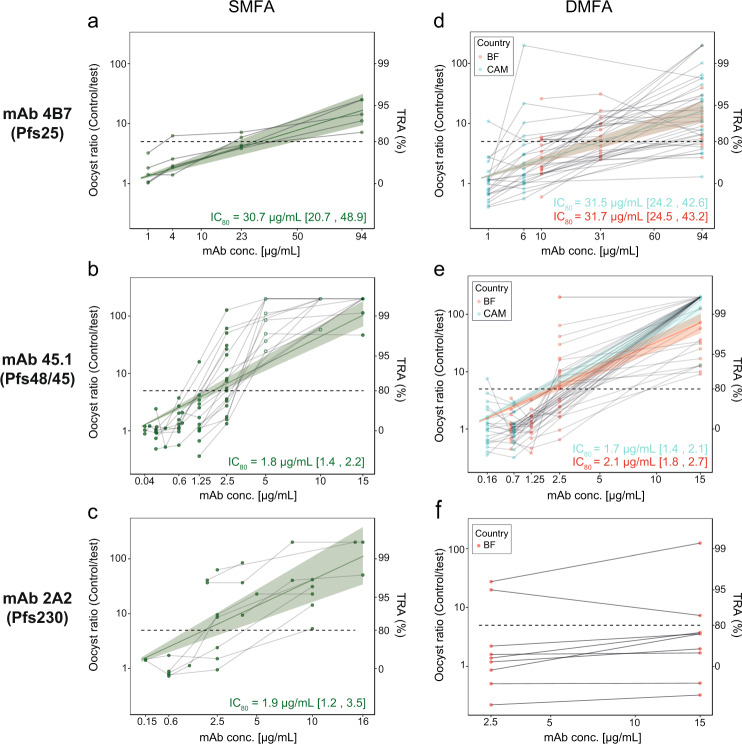
Comparison of transmission-reducing activity of monoclonal antibodies against laboratory-adapted and field strains. **a**–**c** Standard membrane feeding assays (SMFAs) were performed with cultured *P. falciparum* NF54 parasites and *A. stephensi* mosquitoes. Monoclonal antibodies (mAbs) 4B7 (Pfs25, **a**), 45.1 (Pfs48/45, **b**) and 2A2 (Pfs230, **c**) were added at the indicated concentrations to cultured NF54 parasites. Thin grey lines connect observations from individual feeding experiments with multiple mAb concentrations. The green line represents the overall fit from linear regression starting at the intercept (0% TRA at 0 μg/ml). Shaded area represents 95% credible intervals of the fit. mAb concentrations that were not tested in direct membrane feeding assays are omitted from the overall fit (**b**, open circles). **d**–**f** Direct membrane feeding assays (DMFAs) were carried out in Burkina Faso (red) and Cameroon (cyan). Thin grey lines connect observations from individual patients. mAbs 4B7 (Pfs25, **d**), 45.1 (Pfs48/45, **e**) and 2A2 (Pfs230, **f**) were added at the indicated concentrations to venous blood samples. For mAb 2A2 (Pfs230), DMFA was only carried out in Burkina Faso, with two concentrations per donor. The cyan and red line represent the overall fit from linear regression starting at the intercept (0% TRA at 0 μg/ml). Shaded area represents 95% credible intervals of the fit. The mAb concentration is shown on the *x*-axis (square root-transformed). The ratio of oocyst intensity in control conditions over antibody test conditions is shown on the left *y*-axis and corresponding transmission-reducing activity (TRA) on the right *y*-axis. An oocyst ratio of 5 equals 80% TRA, which is indicated by a dashed horizontal line. Note that transmission in some conditions was higher compared to controls, i.e. oocyst ratio <1. It is currently unclear whether this is a methodological artefact or real biological phenomenon related to low antibody density^38^. Calculated IC_80_ values are shown in graphs, with 95% credible intervals shown between brackets. The raw data for DMFA and SMFA experiments can be found in the Supplementary Material (Supplementary Dataset 1 and 2).

Transmission-reducing activity (TRA) was determined by SMFA for three potent mAbs: 45.1 (α-Pfs48/45)^20^, 2A2 (α-Pfs230)^21,22^, and 4B7 (α-Pfs25)^23^. mAbs 45.1 and 2A2 are the most potent transmission-blocking mAbs described to date; 4B7 targets Pfs25 that currently forms the most advanced TBV. All mAbs were raised against *Plasmodium falciparum* NF54. Cultured NF54 parasites were mixed with serial dilutions of mAbs and fed to *Anopheles stephensi* mosquitoes. Oocysts were counted and TRA was calculated as the mean decrease in the number of oocysts per mosquito compared to negative controls from the same experiment^24^. In SMFA experiments, reductions in oocyst intensity (rather than prevalence) are the most robust and informative read-out^25^. All three mAbs showed high TRA against the homologous strain NF54 (Fig. 1a–c). We fitted linear models to the TRA data and estimated mAb concentrations needed to achieve 80% reduction in mean oocyst intensity (IC_80_), a consensus minimum level of TRA to warrant further pre-clinical development^26^. mAbs 45.1 and 2A2 reduced infection intensity in mosquitoes with an IC_80_ of 1.8 μg/mL [95% credible intervals: 1.4, 2.2] and 1.9 μg/mL [1.2, 3.5] respectively, while mAb 4B7 was approximately 16-fold less potent (IC_80_ = 30.7 μg/mL, [20.7, 48.9]).

### Two mAbs reduce transmission in field settings

**Fig. 2:**
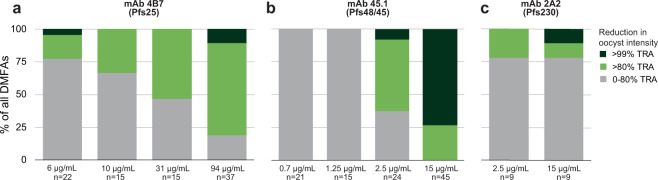
Stratified transmission-reducing activity of three different monoclonal antibodies in direct membrane feeding assays on naturally infected gametocyte carriers. The concentration of **a** monoclonal antibody (mAb) 4B7 (Pfs25), **b** mAb 45.1 (Pfs48/45) and (**c**) mAb 2A2 (Pfs230) in the feeder is indicated below bars. Transmission-reducing activity (TRA) was quantified as the reduction in mean oocyst intensity between antibody control and mAb condition for each donor. Complete transmission-reducing (>99% TRA) is depicted in dark green and strong reduction (80–99% TRA) in bright green. All donors with >1 oocyst/mosquito in antibody control condition (i.e. successful transmission) were included and the number of distinct donor samples (*n*) tested for each antibody is given below bars. mAbs 4B7 and 45.1 were tested at two sites (Burkina Faso and Cameroon) and mAb 2A2 was only tested in Burkina Faso. DMFA direct membrane feeding assays. The raw data for DMFA and SMFA experiments can be found in the Supplementary Material (Supplementary Dataset 1 and 2).

We then tested the efficacy of mAbs in direct membrane feeding assay (DMFA) experiments with blood from naturally infected gametocyte carriers in Cameroon and Burkina Faso. Compared to SMFA, these DMFA experiments more closely resemble natural transmission conditions as they cover variation in gametocyte densities, genetically diverse strains and potentially multiclonal infections. Gametocyte densities were determined for the donors and ranged from 16 to 504 gametocytes/µL (Median: 52; Supplementary Dataset 1). Gametocyte density and average oocyst intensity in DMFA experiments with serum replacement were positively correlated (Spearman’s rank correlation coefficient = 0.67, *p* < 0.0001; Supplementary Fig. 2). Multiplicity of infection (MOI) was determined by AMA1 sequencing for a subset of donors; 12/13 donors carried multiple *P. falciparum* clones (Supplementary Dataset 1). Sequencing of parasite clones was not performed for blood isolates due to complexity of associating genotypes present in the bloodstream in relation to transmission potential^27^. Naturally acquired antibodies were removed by serum replacement and mAbs were added in European control serum at various concentrations before feeding to *Anopheles coluzzii* mosquitoes. At 94 μg/mL, mAb 4B7 oocyst intensity was reduced by >80% in 30/37 of donors and in all (45/45) of donors at 15 μg/mL mAb 45.1. In contrast, at 15 μg/mL mAb 2A2 >80% reduction was only achieved in a minority of the donors (2/9) (Fig. 2). Oocyst prevalence (i.e. the proportion of mosquitoes that became infected) was reduced by >80% after addition of 94 μg/mL mAb 4B7 for 11/37 of donors, for 39/45 donors with 15 μg/mL of mAb 45.1 and for 1/9 of donors with 15 μg/mL mAb 2A2 (Supplementary Fig. 3). We observed comparable IC_80_ values for SMFA and DMFA experiments with mAb 4B7 (Fig. 1a, d) and 45.1 (Fig. 1b, e), demonstrating that these two antibodies reduce transmission of the reference strain and field strains equally well. However, for mAb 2A2 there was a clear discrepancy between SMFA and DMFA; while mAb 2A2 had an IC_80_ of 1.9 μg/mL in SMFA, it showed low TRA at 15 μg/mL in DMFA for most donors with values ranging from −207.5 to 100% (median 72%) (Fig. 1f). We therefore suspected that many patient-derived blood samples contained parasite strains that were not sensitive to mAb 2A2.

### Genetic variation affects transmission-blocking efficacy of Pfs230 mAb 2A2

**Fig. 3:**
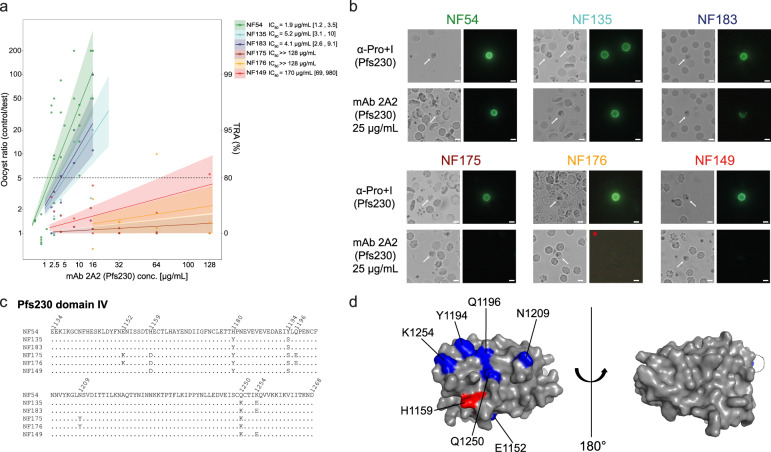
Transmission-reducing activity of monoclonal antibody 2A2 (Pfs230) is strain dependent. **a** Transmission-reducing activity (TRA) of monoclonal antibody (mAb) 2A2 in standard membrane feeding assays with six laboratory-adapted parasite strains from different geographical regions that were fed to *A. stephensi* mosquitoes. Lines represent the fitted linear models and shaded areas the 95% credible intervals. IC_80_ values are shown with 95% credible intervals between brackets. For strains NF175 and NF176, no meaningful IC_80_ values of mAb 2A2 could be obtained due to very low efficacy. The raw data SMFA experiments can be found in the Supplementary Material (Supplementary Dataset 2). **b** Female gamete surface immunofluorescent assay using laboratory-adapted parasite strains incubated with 25 µg/mL of mAb 2A2 or serum from mice that were immunized with a fragment of Pfs230 (Pro+I, amino acids 443–736). Note that NF176 was incubated with 125 µg/mL mAb 2A2 (indicated with red asterisk). White arrows in bright field images indicate activated female gametes. Scale bars represent 10 µm. **c** Amino acid sequence alignment of Pfs230 domain IV (amino acids 1134–1268) of laboratory-adapted parasite strains. Non-synonymous single-nucleotide polymorphism (SNP)-encoded amino acids are specified and invariant amino acids are shown as dots. **d** Surface model of Pfs230 domain IV, based on the crystal structure of Pf41 (Pf3D7_0404900; PDB id: 4YS4), highlighting amino acids that vary among the tested strains. Histidine 1159 (H1159) is mutated to aspartate in all strains that are not sensitive to mAb 2A2 and is highlighted in red. Other amino acid variants are shown in blue. Histidine 1180 (H1180) is located in a non-structured loop, which is presented as a dashed line.

To further investigate the impact of genetic variation on the efficacy of mAb 2A2, SMFA experiments with several laboratory-adapted parasite strains were performed. SMFAs with six *P. falciparum* strains demonstrated variation in antibody efficacy of mAb 2A2 (Fig. 3a) in line with the observed variation in the DMFA (Fig. 1f). Transmission of the reference strain NF54 was effectively blocked, while for NF135 and NF183 slightly higher concentrations were needed to achieve >80% TRA (Fig. 3a). Strains NF175, NF176 and NF149 were not sensitive to mAb 2A2, even at the highest antibody concentrations tested.

Binding experiments in which female gametes were incubated with mAb 2A2 demonstrated a similar heterogeneity in antibody binding to parasite strains (Fig. 3b and Supplementary Fig. 4). Strong reactivity of the mAb with native protein on the gamete surface of NF54 parasites was observed, even at a low concentration of 0.2 µg/mL (Supplementary Fig. 4). Reactivity with the surface of NF135 and NF183 gametes was observed at 25 µg/mL (Fig. 3b), but not at concentrations <1 µg/mL (Supplementary Fig. 4). Strikingly, no antibody reactivity was observed with strains NF175, NF176 and NF149 (Fig. 3b). For the strains that showed binding with mAb 2A2 at all tested concentrations, we did not detect any non-specific signal with the secondary antibody (Supplementary Fig. 4). Binding of mAb 2A2 is thus in line with its TRA in SMFA. The observed differences in antibody binding were not the result of variation in Pfs230 antigen abundance as demonstrated by binding experiments with polyclonal anti-Pfs230 mice serum (Fig. 3b), suggesting that polymorphisms affect binding and thereby cause differences in functional activity.

The Pfs230 domain that is targeted by mAb 2A2 is currently unknown. In western blots, mAb 2A2 did not recognize recombinantly expressed fusion of pro-domain and domain I, while it did recognize Pfs230 in gametocyte extract (Supplementary Fig. 5). We explored *Pfs230* sequence data to identify the antibody-binding site. We had full genome sequences available for three of the strains and full-length *Pfs230* gene sequences revealed five domains that contain unique non-synonymous single-nucleotide polymorphisms (SNPs) for NF175 that are not present in NF54 and NF135 (Supplementary Fig. 6). Sequencing of these five domains in the other three strains suggested that domain IV contains the presumed binding site of mAb 2A2, since this is the only domain that contains non-synonymous SNPs in all three non-sensitive strains (NF149, NF175 and NF176) that are absent in the sensitive strains (Fig. 3c and Supplementary Fig. 7). One non-synonymous SNP, resulting in an H1159D mutation, is unique to non-sensitive strains, suggesting that this residue could be critical for antibody binding. However, other identified non-synonymous SNPs may further affect binding, explaining the lower antibody efficacy against NF135 and NF183 compared to NF54. A three-dimensional (3D) model of domain IV revealed that most of these non-synonymous SNP-encoded amino acids are in close proximity to each other, and it is therefore plausible that several are located in the mAb 2A2-binding site (Fig. 3d).

The amino acid variations that we find in Pfs230 domain IV in the laboratory strains are also observed in parasite strains from most geographical regions^28^ indicating that there is no geographical fixation (Supplementary Fig. 8). Analysis of all known non-synonymous SNPs in *Pfs230* as reported by PlasmoDB v46 demonstrates that domain IV is the most polymorphic region of the protein (Supplementary Fig. 9). Altogether, our data strongly suggest that domain IV is the binding domain of mAb 2A2 and that amino acid mutations affect antibody binding and thereby efficacy.

### Frequency of non-synonymous SNPs is not associated with antibody pressure

**Fig. 4:**
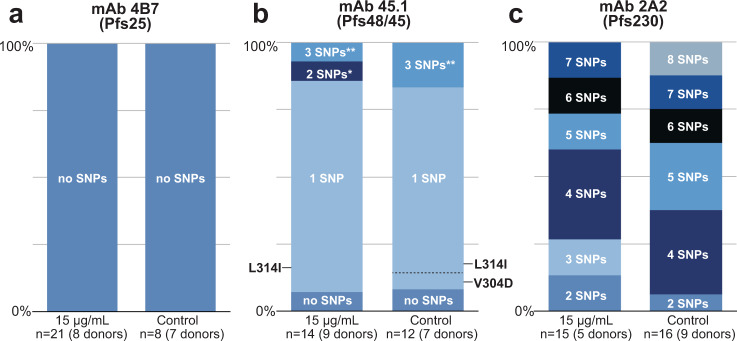
Genotypes of single oocysts that formed in the presence of high concentrations of monoclonal antibodies. Oocyst genotypes from direct membrane feeding experiments with high concentrations of mAb 4B7 (Pfs25), mAb 45.1 (Pfs48/45) and mAb 2A2 (Pfs230) and from a control condition without mAb were determined. The sequences were compared to the NF54 reference strain to identify non-synonymous single-nucleotide polymorphisms (SNPs). Regions that were analyzed were full-length Pfs25 (**a**), 6C domain of Pfs48/45 (**b**) and domain IV of Pfs230 (**c**). Only oocysts from Burkina Faso were available and included in this analysis. Single oocysts were not available for all donors or conditions, the ‘*n*’ represents the number of oocysts analysed per condition with the number of donors in brackets. The raw data of this figure can be found in the Supplementary Material (Supplementary Tables 2–4). *SNPs L314I and V304D; **SNPs L314I, V304D and S322N.

Based on variation in activity of mAb 2A2 in SMFA and DMFA, we hypothesized that oocysts which formed despite high concentrations of mAb were more likely to contain non-sensitive gene variants. To test this hypothesis, we extracted DNA from single oocysts that formed in mosquitoes that fed on gametocytes in the presence of high mAb concentrations and sequenced the targeted domains. These data were available for donors from Burkina Faso only, where single oocyst dissections were performed. Oocyst DNA was also sequenced from control samples without test antibodies. We did not find non-synonymous SNPs in *Pfs25* in oocysts from both the antibody and control condition (Fig. 4a and Supplementary Table 1).

Five non-synonymous SNPs have previously been reported in the 6C domain of Pfs48/45, which is targeted by mAb 45.1 (PlasmoDB.org v46^29^). We detected three of these among the sequenced oocysts (Fig. 4b and Supplementary Table 2). The most frequent amino acid change (L314I), with a reported minor allele frequency of 40%, was present in 13/14 oocysts isolated from mosquitoes that received a blood meal with high mAb 45.1 concentrations and in 9/13 antibody control oocysts. All three non-synonymous SNPs are distal to the previously characterized binding site of mAb 45.1 and thus unlikely to affect binding (Supplementary Fig. 10). A minority of oocysts contained the NF54 sequence (1/14 high mAb oocysts and 3/13 antibody control oocysts; Fig. 4b). For some donors, we sequenced multiple oocysts and obtained more than one unique sequence, as could be expected from multiclonal infections.

For Pfs230, we examined the sequence of domain IV, which we identified as the likely target of mAb 2A2. No exact NF54 reference sequence was observed. Two to eight non-synonymous SNPs were detected in every oocyst examined (*n* = 15 for 15 μg/mL mAb 2A2 and *n* = 16 for control; Fig. 4c; Supplementary Table 3). At three positions (amino acid mutations H1180Y, Y1194S and Q1250K), NF54 carries the minor allele while most oocysts and all five analyzed laboratory-adapted strains carry the major allele (Fig. 2c). The eight non-synonymous SNPs observed in the oocysts were reported before and exact combinations of these SNPs for each oocyst are diverse (Supplementary Table 3). Five oocysts were identical to the NF135 genotype, but none of the other 31 oocysts matched with any of our laboratory strains. No clear difference was observed in the number or combination of non-synonymous SNPs in oocysts that appeared at high mAb 2A2 concentration compared to control condition.

## Discussion

One of the major hurdles in malaria vaccine and antibody development is genetic variation across *Plasmodium* strains. Since mAbs target single epitopes, they are particularly vulnerable to polymorphisms. Although sexual stage antigens are more conserved than asexual stage antigens, it is unknown whether transmission-blocking antibodies indeed provide cross-strain protection. In in vitro experiments with *P. falciparum* strains and ex vivo assessments of mAb potency against naturally acquired gametocytes, we demonstrate considerable variation in functional activity between three potent transmission-blocking mAbs that target antigens that are currently considered for vaccine development.

mAbs are now increasingly considered as interventions for malaria since potent human(ized) anti-*Plasmodium* mAbs are available^30,31^ and mAbs are becoming affordable for use in low-income settings^32^. mAbs that block transmission to mosquitoes can be powerful tools to reduce transmission in areas aiming for the elimination of (drug-resistant) malaria and areas characterized by short seasonal malaria transmission. We evaluated the potency of three established transmission-blocking mAbs against naturally acquired *P. falciparum* strains in Cameroon and Burkina Faso and compared to the potency against the strain they were raised against (NF54). mAb 45.1 showed similar potency in field-based DMFA compared to SMFA with NF54, demonstrating that it efficiently blocks genetically diverse parasite strains and multiclonal infections. While the overall potency of mAb 45.1 was similar, we sporadically observed oocysts at high mAb concentrations in DMFA (Fig. 1b, e). Since many of our donors carried multiclonal *P. falciparum* infections, introduction of the mAb could potentially block sensitive clones but permit transmission of concurrently present mutant clones. To test this hypothesis, we sequenced the *Pfs48/45-6C* locus of single oocysts that appeared at high concentrations of mAb 45.1 to determine whether these are enriched for certain parasite genotypes as compared to control feeds. Although the number of oocysts that were available was too low for meaningful statistical comparisons, we observed no clear enrichment of ‘escape’ variants (Fig. 4b). In total, we identified three previously described SNPs^33^, all of which are outside the mAb 45.1-binding site and thus unlikely to abrogate binding (Supplementary Fig. 10). Moreover, at high mAb 45.1 concentration we identified one oocyst that had an identical sequence of the 6C domain as NF54. Taken together, it seems unlikely that parasites escape antibody pressure due to polymorphisms in the 6C domain. Small differences in achieving full transmission blockade between DMFA and SMFA while having highly comparable IC_80_ estimates have to be interpreted with caution and may be a chance finding in the limited number of individual experiments. The current data do not indicate substantial heterogeneity in mAb efficacy. Similar findings were obtained for mAb 4B7. Although this mAb had considerably lower potency, it also blocked transmission of genetically diverse parasite strains in DMFA, as was previously suggested for another Pfs25-targeting mAb^34^ and for sera from volunteers immunized during a Pfs25/ISA51 phase 1 trial^35^.

In contrast, the Pfs230 targeting mAb 2A2 failed to block transmission of gametocytes from most gametocyte donors tested. While this was based on a limited number of experiments and it is not possible to estimate what fraction of naturally acquired infections was not blocked by 2A2, the limited mAb efficacy is striking. This contrasts with its potency in the SMFA and findings with polyclonal antibodies^34,36^ and strongly suggests that genetic variation in the *Pfs230* locus of circulating gametocytes affects the efficacy of this mAb. Using six laboratory-adapted strains from geographically distinct locations, we demonstrated that mAb 2A2 did not bind to the gamete surface of certain strains and that binding was in line with functional TRA. The target epitope of mAb 2A2 is currently unknown. mAb 2A2 did not recognize a properly folded recombinant protein that covers domain I and the pro-domain (Supplementary Fig. 5); sequence data from our six laboratory-adapted strains highlighted domain IV of Pfs230, the domain with the highest SNP density of all Pfs230 domains (Supplementary Fig. 9), as plausible target.

Importantly, many immunogenicity studies with recombinant Pfs230 fragments have demonstrated that only antibodies induced against the pro-domain and domain I of Pfs230, which together encompass the leading vaccine construct D1M^37^, block transmission. Our data strongly suggest that another domain of Pfs230 can also be the target of potent transmission-blocking antibodies. Recombinant fragments covering domains outside the pro-domain and domain I may have failed to induce efficacious antibodies in previous studies due to improper folding of recombinant immunogens. Our data support renewed efforts to produce properly folded Pfs230 fragments, which could lead to the discovery of potent novel Pfs230-based vaccine candidates.

In conclusion, we demonstrate that evaluating potency of transmission-blocking mAbs against field strains is important in the (pre-)clinical development process and for identification of potent and conserved epitopes to inform vaccine design and development. We tested three different mAbs against three leading TB vaccine candidates and found that the potency of one of these was affected by genetic diversity in contrast to the other two mAbs. Increased oocyst densities were sporadically observed in experiments with low mAb concentrations. Whether this is explained by biologically relevant transmission enhancement or methodological issues is currently unknown^38^ and requires dedicated studies. Our data are not predictive for cross-strain efficacy of other mAbs and polyclonal antibodies to these targets as other antibodies may target different epitopes. Our data suggest that mAb2A2 targets domain IV, which is the most genetically diverse domain of Pfs230 (Supplementary Fig. 9). The pro-domain and domain I contained in the leading Pfs230 vaccine construct (D1M) are more conserved and mAbs and polyclonal antibodies against this target are therefore more likely to provide cross-strain protection. Indeed, a potent human mAb (LMIV230-01) identified in immunized volunteers recognizes and fixes complement on geographically distinct strains^39^ and Pfs230D1M-based vaccines show great promise^14^. The most potent human mAbs against Pfs230 and Pfs25 are LMIV230-01 (IC_80_ = 10–60 µg/mL) and mAb2544 (IC_80_ = 16 µg/mL)^40^ and more potent mAbs may be discovered in the future.

Importantly, mAb 45.1 is the most potent transmission-blocking mAb described to date and shows potent cross-strain protection in our DMFA experiments. A fully humanized version of mAb 45.1, TB31F^30^, is currently being tested in a phase I experimental medicine study (clinicaltrials.gov: NCT04238689). Based on our equivalent SMFA and DMFA data for mAb 45.1, it can be assumed that SMFA data obtained from this clinical trial will be predictive for efficacy in field settings. Importantly, our data show that mAb 45.1 targets a conserved protective epitope, which may be used in the design of Pfs48/45-based vaccines to specifically induce antibodies against this epitope. Potent human mAbs may be obtained from scheduled phase I vaccination trials with Pfs48/45. Here we provide a framework for testing these newly identified mAbs against genetically diverse parasites to further inform design of this leading TBV candidate.

## Methods

### mAb production

The generation of the mAbs against Pfs48/45^20^, Pfs230^21,22^ and Pfs25^23^ have been described previously. Briefly, Pfs230 mAb 2A2.2a has been derived from mice immunized with *P. falciparum* NF54 gametocyte extracts and underwent isotype switch to IgG2a as described previously^21,22^. The Pfs48/45 targeting mAb 85RF45.1 (IgG1) has been derived from rats immunized with NF54 gametocyte extracts^20^. Pfs25 mAb 4B7 (IgG1) has been derived from mice immunized with recombinant vaccinia virus expressing Pfs25^23^. mAb 45.1 and 2A2 antibodies were affinity purified from clarified hybridoma supernatant using MabSelect Xtra™ and HiTrap Protein G columns (GE Healthcare Life Sciences), respectively. Elution fractions containing the IgGs were dialysed against phosphate-buffered saline (PBS), concentrated and freeze-dried for storage. They were dissolved in distilled water before use. mAb 4B7 was a kind gift from PATH Malaria Vaccine Initiative.

### Laboratory-adapted parasite strains and culture

*P. falciparum* strains were isolated from malaria patients and were adapted to culture as described previously^41^. Briefly, parasites were cultured in RPMI1640 medium supplemented with 10% human serum at 5% haematocrit in a semiautomated suspension culture system and cloned by limited dilution. The West-African NF54^42^, Cambodian NF135.C10^43^ and Nigerian NF175.D5^44^ parasite strains have been described previously and compared at the genome level^45^. Furthermore, NF149.A3 (Southern-Asia), NF176.B8 (East-Africa) and NF183.F7 (West-Africa) were used in this study^46^. The clonality and identity of parasite strains was confirmed using polymerase chain reaction (PCR) on glutamate-rich protein and merozoite surface proteins 1 and 2 (MSP1 and MSP2) as described previously^47^.

### Standard membrane feeding assay

SMFAs were performed as described previously^48^. In brief, *A. stephensi* mosquitoes from a colony maintained at Radboudumc (Nijmegen, NL) were fed with *P. falciparum* mature gametocyte cultures (days 15–17) mixed with human serum and active human complement. mAbs were added to the gametocytes to achieve the indicated final concentrations in the offered blood meals. Mosquito midguts from a minimum of 20 mosquitoes per condition were dissected, stained with mercurochrome and checked for the presence of oocysts 6–8 days after the blood meal.

### Naturally infected gametocyte donors

Asymptomatic gametocyte carriers, aged 5–15 years, were enrolled in Mfoe district (Cameroon) and Bobo Dioulasso (Burkina Faso). Venous blood samples of 29 donors in Cameroon and 30 donors in Burkina Faso were collected after written informed consent was obtained from participants or their guardian(s). Ethical approval was provided by the National Ethics Committee of Cameroon; Ethical Review Committee of the Ministry of Health, Burkina Faso; Institutional ethics review committee for health science research Bobo Dioulasso; University of California, San Francisco; and London School of Hygiene and Tropical Medicine. Participant characteristics of these cohorts have been described previously^49^.

### Direct membrane feeding assay

Patient blood containing gametocytes was collected in heparin tubes. Per tested condition, 500 µL of heparinized blood was centrifuged (5 min at 2000 × *g*) and plasma was removed, then replaced by 250 µL naive serum (=antibody control) or by naive serum pre-mixed with mAb, achieving the final concentrations of mAb in the total 500 µL volume. DMFA experiments that included testing of mAb 2A2 were done with normal human serum, which contains active complement, from naive European donors. In each DMFA experiment, at least two mAbs were tested in parallel. Minifeeders were filled with the mixture and *A. coluzzii* mosquitoes from local mosquito colonies were allowed to feed for 20 min. All materials were kept at 37 °C during the experiment. Fully fed mosquitoes were kept 6–8 days post feeding; unfed and partially fed mosquitoes were removed and not included in the analyses. Approximately 20–30 mosquitoes were dissected for each condition, midguts were stained with mercurochrome and oocysts were counted. During dissection, infected midguts were collected and stored in absolute ethanol at −80 °C. Subsequently, a selection of midguts was gradually rehydrated in distilled water and single oocysts were isolated according to the method described by Annan et al.^50^. These single oocyst dissections were performed in Burkina Faso only. The single oocysts were stored in RNAprotect (Qiagen®, Hilden, Germany) at −80 °C.

### Surface immunofluorescent assay (SIFA)

Cultured gametocytes (days 15–17) were activated by incubation in foetal calf serum (FCS) for 1 h at room temperature. Cells that include gametes were washed with PBS and incubated with mAb 2A2 dilutions in SIFA buffer (PBS/0.5% FCS/0.05% sodium azide). The presence of Pfs230 on gamete surfaces was confirmed by incubation with 1:20 dilution of serum from mice immunized with Pfs230 fragment Pro+I (amino acids 443–736)^51^. Gamete preparations were washed three times with SIFA buffer and incubated with 1:200 Alexa Fluor® 488 Goat Anti-Mouse IgG (H+L) (Invitrogen, Cat. No. A11001) at a 1:200 dilution in SIFA buffer. All incubation steps were performed on ice for 1 h. For each antibody concentration, three individual gametes were imaged and representative pictures are shown.

### Pfs230 domain IV model

The sequence of domain IV (amino acids E1134–D1268) was used to generate a 3D model using the Fold & Function Assignment Server (FFAS) of the Godzik laboratory^52^. The best hit in the PDB database (PDB1018) was Pf41 (Pf3D7_0404900; PDB id: 4YS4). Based on the alignment of the two sequences and the structure of Pf41, a model was generated using default settings (SCWRL modelling method and All-atom model type). Figures were generated with PyMOL (Molecular Graphics System Version 2.3 Schrödinger, LLC).

### Target antigen sequencing

DNA from single oocysts was isolated using phenolchloroform (PC) extraction, following a slightly modified protocol published by Ranford-Cartwright et al.^53^. In brief, the oocyst and surrounding mosquito midgut material were digested overnight at 56 °C in oocyst lysis buffer containing proteinase K. PC extraction was followed by isopropanol precipitation and the obtained pellet was washed with 70% ethanol, dried and dissolved in 20 μL H_2_O. The target sequences were amplified from the obtained genomic DNA by nested or semi-nested PCRs. In some cases, mixed signals were obtained. These were either caused by the presence of multiple oocysts or single oocysts that were a product of a male and female gamete with different genotypes. These mixed oocysts were counted as non-synonymous SNPs.

For sequence analysis of *Pfs230* from cultured parasite strains, genomic DNA was isolated from mixed parasite cultures using the QIAamp® DNA Blood Mini Kit (Qiagen®, Hilden, Germany). The *Pfs230* gene (domains I–XIV) was amplified by PCR using PrimeSTAR GXL DNA Polymerase (TaKaRa Bio, Shiga, Japan) according to the manufacturer’s instructions. Domains that were sequenced were selected based on the observed phenotypes of NF54, NF135 and NF175 in the SMFA and the previously available sequence data of these strains (Supplementary Fig. 6). Mutations were confirmed in duplicate PCR reactions and two independent sequencing reads per PCR reaction.

Sanger sequencing was performed at BaseClear (Leiden, the Netherlands), and data were analyzed using Chromas Lite (Version 2.1.1, Technelysium Pty., Australia). Primers for amplification and sequencing are listed Supplementary Table 4.

### In silico sequence variation analysis

The available Pfs230 (PF3D7_0209000) protein sequences were downloaded from PlasmoDB^29^. The domain composition as defined in Gerloff et al.^54^ was used to calculate the density of polymorphisms per region. For the distribution of the polymorphisms in domain IV over various regions, we used version 6 of the MalariaGEN *P. falciparum* Community Project that contains VCF files of 7113 sequenced strains^28^. Per strain, we included the most frequently detected variant. Nucleotide changes were translated into proteins and the sequence variation per region was visualized using the sequence logo generator WebLogo version 2.8.2^55^ after performing a multiple sequence alignment using Clustal Omega^56^.

### Multiplicity of infection

Parasite material (total nucleic acids isolated from whole blood) from study participants was analyzed by amplicon deep sequencing of apical membrane antigen 1 (AMA-1). Haemi-nested PCR was used to amplify a 236 base-pair segment of AMA-1 using a published protocol^57^. Samples were amplified in duplicate, indexed, pooled and purified by bead cleaning. Sequencing was performed on an Illumina MiSeq platform (150 bp paired-end). Data extraction, processing and haplotype clustering were performed using SeekDeep^58^. MOI was calculated as the number of haplotypes present in both replicates of a given sample.

### Statistical analysis of the membrane feeding assay data

TRA was quantified as the relative reduction in oocyst intensity (mean oocyst count per dissected mosquito) when using mAbs compared to the serum replacement (no antibody control) condition. We quantified transmission reduction as suggested by Miura et al.^24^. We fitted linear models to the TRA data by modelling the relationship between the (log transformed) ratio of the oocyst counts observed in the control and test arms and the (square root transformed) mAb concentration. When oocysts with added mAb are completely absent or at least 200-fold lower than that in the control, we assumed a 1:200 ratio (99.5% TRA). As we assumed zero TRA (i.e. an oocyst ratio of one) in the absence of antibodies, we did not allow the intercept of the regression models to vary when fitting the models. For each antibody, we began with the simplest model possible, using the same slope for the DMFA and SMFA data. No model was fit for the DMFA data of 2A2, because of the heterogeneity of the results. We then developed more complicated models and examined whether the model fit improved. Using weakly informative priors, we allowed the slopes to vary for the assay used and, for the DMFA data, the country from which parasites were collected. We also included a random effect in the slope for either the donor (DMFA data) or the experiment (SMFA data). The regression modelling was carried out using RStan (package version 2.21.1)^59^ and rethinking (package version 2.01)^60^, and the goodness of fit was assessed via the widely applicable information criterion^61^. For mAb 45.1, allowing the slopes to vary by assay and country slightly improved the goodness of fit. For mAb 4B7, the more complicated models did not outperform the simpler model (the same average slope for all the data). For the results shown in Fig. 1, we present model predictions from an ensemble of models, generated using the Akaike weight associated with each model^61^. In the case of mAb 4B7, for example, the models for the DMFA data from Burkina Faso and Cameroon are nearly identical, as not much weight was attributed to models that contained the country-specific term. For mAb 45.1, the country-specific differences are more apparent but still quite small. All models and respective goodness of fit are summarized in Supplementary Table 5. The slopes of the regression models fitted to the SMFA data of mAb 2A2 against the laboratory-adapted strains are provided in Supplementary Table 6. SMFA and DMFA feeds were excluded from the analysis for TRA if control mosquitoes had on average <1 oocyst/mosquito or <30% of mosquitoes became infected, with the exception of SMFA data for NF149 since transmission of this line is in general less efficient. The raw data for DMFA and SMFA experiments can be found in the Supplementary Material (Supplementary Dataset 1 and 2).

### Reporting summary

Further information on research design is available in the Nature Research Reporting Summary linked to this article.

## Supplementary information

Reporting Summary

Supplementary Files

Supplementary Dataset 1

Supplementary Dataset 2

## Data Availability

All data generated and analyzed during this study are included in this published article and its Supplementary Information files. Nucleotide sequences of Pfs230 domain IV from all six laboratory-adapted *P. falciparum* strains are available under GenBank accession numbers MZ517163–MZ517168. All unique materials described in this paper, including parasite isolates, are available upon reasonable request.

## Code availability

The R scripts and input files that were used to perform the regression analysis and generate Figs. 1 and 3a are available on Github: https://github.com/JDChallenger/malaria_monoclonal_transmission.
