# Supplementary material for: Monoclonal antibodies block transmission of genetically diverse Plasmodium falciparum strains to mosquitoes
Source: NPJ Vaccines. 2021 Aug 12;6:101. doi: 10.1038/s41541-021-00366-9 (PMC8361195; doi:10.1038/s41541-021-00366-9)
Supplement: Supplementary file 1 — Reporting Summary [file 41541_2021_366_MOESM1_ESM.pdf]

nature research

Corresponding author(s): Teun Bousema & Matthijs M. Jore

Last updated by author(s): Jun 8, 2021

Reporting Summary

Nature Research wishes to improve the reproducibility of the work that we publish. This form provides structure for consistency and transparency in reporting. For further information on Nature Research policies, see our Editorial Policies and the Editorial Policy Checklist.

Statistics

For all statistical analyses, confirm that the following items are present in the figure legend, table legend, main text, or Methods section.

n/a	Confirmed	
☐	☒	The exact sample size (n) for each experimental group/condition, given as a discrete number and unit of measurement
☐	☒	A statement on whether measurements were taken from distinct samples or whether the same sample was measured repeatedly
☒	☐	The statistical test(s) used AND whether they are one- or two-sided<br>*Only common tests should be described solely by name; describe more complex techniques in the Methods section.*
☒	☐	A description of all covariates tested
☒	☐	A description of any assumptions or corrections, such as tests of normality and adjustment for multiple comparisons
☒	☐	A full description of the statistical parameters including central tendency (e.g. means) or other basic estimates (e.g. regression coefficient) AND variation (e.g. standard deviation) or associated estimates of uncertainty (e.g. confidence intervals)
☒	☐	For null hypothesis testing, the test statistic (e.g. F, t, r) with confidence intervals, effect sizes, degrees of freedom and P value noted<br>*Give P values as exact values whenever suitable.*
☐	☒	For Bayesian analysis, information on the choice of priors and Markov chain Monte Carlo settings
☒	☐	For hierarchical and complex designs, identification of the appropriate level for tests and full reporting of outcomes
☒	☐	Estimates of effect sizes (e.g. Cohen's d, Pearson's r), indicating how they were calculated

Our web collection on statistics for biologists contains articles on many of the points above.

Software and code

Policy information about availability of computer code

Data collection	No software was used for data collection
Data analysis	Data analysis carried out in R using RStan (package version 2.21.1) and rethinking (package version 2.01). R scripts and data input files are provided as a supplementary zip file.

For manuscripts utilizing custom algorithms or software that are central to the research but not yet described in published literature, software must be made available to editors and reviewers. We strongly encourage code deposition in a community repository (e.g. GitHub). See the Nature Research guidelines for submitting code & software for further information.

Data

Policy information about availability of data

All manuscripts must include a data availability statement. This statement should provide the following information, where applicable:

- Accession codes, unique identifiers, or web links for publicly available datasets
- A list of figures that have associated raw data
- A description of any restrictions on data availability

All data generated and analyzed during this study are included in this published article and its supplementary information files.

Field-specific reporting

Please select the one below that is the best fit for your research. If you are not sure, read the appropriate sections before making your selection.

☒ Life sciences ☐ Behavioural & social sciences ☐ Ecological, evolutionary & environmental sciences

For a reference copy of the document with all sections, see nature.com/documents/nr-reporting-summary-flat.pdf

Life sciences study design

All studies must disclose on these points even when the disclosure is negative.

Sample size	Sample size was based on prior experience in our study site in Burkina Faso where ex vivo assessments of transmission-reducing activity of polyclonal antibodies with 21 donors with identical parasitological characteristics (>2 gametocytes/500WBC) allowed precise estimates of TRA across antibody concentrations (https://www.nature.com/articles/s41598-017-06130-1.pdf)
Data exclusions	Only results from SMFA and DMFA feeds were excluded from analysis for transmission reducing activity if control mosquitoes had on average less than one oocyst/mosquito or less than 30% of mosquitoes were infected. This is specified in the methods. Excluded data are included in Supplementary Tables 1 and 2
Replication	Presented data are based on multiple DMFA (with different donors) or SMFA experiments for each antibody condition. Gamete staining experiments were conducted twice to confirm findings.
Randomization	No randomization was applied; different mAbs and concentrations of mAbs were tested with gametocytes from the same donor.
Blinding	Specific blinding was not used because oocyst counting data for DMFA/SMFA experiments is not subjective.

Reporting for specific materials, systems and methods

We require information from authors about some types of materials, experimental systems and methods used in many studies. Here, indicate whether each material, system or method listed is relevant to your study. If you are not sure if a list item applies to your research, read the appropriate section before selecting a response.

Materials & experimental systems

n/a	Involved in the study	
☐	☒	Antibodies
☐	☒	Eukaryotic cell lines
☒	☐	Palaeontology and archaeology
☐	☒	Animals and other organisms
☐	☒	Human research participants
☒	☐	Clinical data
☒	☐	Dual use research of concern

Methods

n/a	Involved in the study	
☒	☐	ChIP-seq
☒	☐	Flow cytometry
☒	☐	MRI-based neuroimaging

Antibodies

Antibodies used	Non-commercial mAbs (2A2, 4B7, 45.1 and 15C5) are described in the methods section and include references to the original papers. For gamete staining experiments Alexa Fluor 488 Goat Anti-Mouse IgG (H+L) (Invitrogen, Cat. A11001, Lot. 1907294) was used. For western blots Goat Anti-human IRDye680RD (LI-COR, Cat. 926-68078, Lot. C90108-04) and Goat Anti-Mouse IRDye680RD (LI-COR, Cat. 926-68070, Lot. C41009-01) were used.
Validation	Antigen-specificity for non-commercial antibodies was confirmed by western blot. LI-COR antibodies were tested by dot blot and/or solid-phase adsorbed to ensure minimal cross-reactivity by manufacturer. Alexa-Fluor 488 Goat Anti-Mouse antibody is an affinity purified antibody from Invitrogen.

Eukaryotic cell lines

Policy information about cell lines

Cell line source(s)	Clinical isolates of Plasmodium falciparum were adapted to culture and cloned at Radboudumc
Authentication	Clonality and identity of each line was confirmed by PCR on hypervariable genes (GLURP, MSP1 and MSP2)
Mycoplasma contamination	N/A

Commonly misidentified lines (See ICLAC register)	N/A

Animals and other organisms

Policy information about studies involving animals; ARRIVE guidelines recommended for reporting animal research

Laboratory animals	Anopheline mosquitoes from local colonies were used in this study.
Wild animals	N/A
Field-collected samples	N/A
Ethics oversight	no ethics approval is required for work with mosquitoes from colony

Note that full information on the approval of the study protocol must also be provided in the manuscript.

Human research participants

Policy information about studies involving human research participants

Population characteristics	Asymptomatic gametocyte carriers, aged 5-15 years, were enrolled in Mfoe district (Cameroon) and Bobo-Dioulasso (Burkina Faso).
Recruitment	Membrane feeding assays were performed on asymptomatically infected individuals who were recruited in community surveys. Blood was drawn prior to treatment and after written informed consent was obtained from participants or their guardian(s).
Ethics oversight	Ethical approval was provided by the National Ethics Committee of Cameroon; Ethical Review Committee of the Ministry of Health, Burkina Faso; Institutional ethics review committee for health science research Bobo-Dioulasso; University of California, San Francisco, and London School of Hygiene and Tropical Medicine.

Note that full information on the approval of the study protocol must also be provided in the manuscript.
