# Supplementary material for: Monoclonal antibodies block transmission of genetically diverse Plasmodium falciparum strains to mosquitoes
Source: NPJ Vaccines. 2021 Aug 12;6:101. doi: 10.1038/s41541-021-00366-9 (PMC8361195; doi:10.1038/s41541-021-00366-9)
Supplement: Supplementary file 2 — Supplementary Files [file 41541_2021_366_MOESM2_ESM.pdf]

Supplementary Table 1. Non-synonymous SNPs in *Pfs25* in sequenced oocysts

Sample ID	SNPs	Donor ID	mAb 4B7 condition	MOI
48	None	BF17.004	Control	ND
7	None	BF17.006	Control	ND
13	None	BF17.010	Control	10
56	None	BF17.013	Control	7
47	None	BF17.013	Control	7
54	None	BF17.014	Control	ND
52	None	BF17.015	Control	ND
L07	None	BF17.020	Control	ND
L14	None	BF17.002	94 µg/mL	7
L13	None	BF17.002	94 µg/mL	7
2	None	BF17.002	94 µg/mL	7
3	None	BF17.002	94 µg/mL	7
L12	None	BF17.003	94 µg/mL	15
L10	None	BF17.003	94 µg/mL	15
L16	None	BF17.003	94 µg/mL	15
5	None	BF17.003	94 µg/mL	15
6	None	BF17.003	94 µg/mL	15
8	None	BF17.006	94 µg/mL	ND
9	None	BF17.006	94 µg/mL	ND
L15	None	BF17.007	94 µg/mL	ND
11	None	BF17.007	94 µg/mL	ND
14	None	BF17.010	94 µg/mL	10
15	None	BF17.010	94 µg/mL	10
17	None	BF17.015	94 µg/mL	ND
18	None	BF17.016	94 µg/mL	ND
20	None	BF17.016	94 µg/mL	ND
L04	None	BF17.016	94 µg/mL	ND
L05	None	BF17.016	94 µg/mL	ND
22	None	BF17.018	94 µg/mL	12

We detected two non-synonymous SNPs that were not reported before in any database. We therefore repeated the amplification and sequencing of these oocysts but retrieved NF54 reference sequences. These oocysts were therefore excluded from this analysis. ND: Not determined; MOI: Multiplicity of infection.

Supplementary Table 2. Non-synonymous SNPs in *Pfs48/45-6C* in sequenced oocysts

	V304D	L314I	S322N			
Sample ID	ref T, alt A	ref T, alt A	ref G, alt A	Patient ID	mAb 45.1 condition	MOI
23	T	A	G	BF17.001	Control	3
1	T	T	G	BF17.001	Control	3
3**	A/T	A/T	A/G	BF17.002	Control	7
6**	T	A/T	G	BF17.003	Control	15
48*	T	A	G	BF17.004	Control	ND
8	T	A	G	BF17.004	Control #	ND
11	A/T	A	A/G	BF17.006	Control	ND
13#	T	A	G	BF17.006	Control	ND
50	T	A	G	BF17.006	Control	ND
49	A/T	T	G	BF17.008	Control	ND
44	T	A/T	G	BF17.009	Control	ND
43	T	A	G	BF17.009	Control	ND
9	A/T	A	A/G	BF17.004	15 µg/mL	ND
25	T	A	G	BF17.004	15 µg/mL	ND
29	T	A	G	BF17.011	15 µg/mL	ND
30	T	A	G	BF17.012	15 µg/mL	3
32	T	A	G	BF17.015	15 µg/mL	ND
34	T	A	G	BF17.015	15 µg/mL	ND
14	T	T	G	BF17.016	15 µg/mL	ND
17*	A/T	A/T	G	BF17.017	15 µg/mL	ND
16*	T	A	G	BF17.017	15 µg/mL	ND
18	T	A	G	BF17.019	15 µg/mL	ND
19	T	A	G	BF17.019	15 µg/mL	ND
20	T	A	G	BF17.019	15 µg/mL	ND
21*	T	A/T	G	BF17.021	15 µg/mL	ND
B07	T	A/T	G	BF18.002	15 µg/mL	5

Occasionally, two different nucleotides are mentioned. These are either due to the presence of multiple oocysts or, single 'mixed' oocysts, derived from the gametes of two distinct clones present in the respective donor (MOI>1). These mixed signal oocysts were counted as non-synonymous SNPs.

* Sample contained two oocysts
** Sample contained three oocysts
\# Oocysts from mAb 4B7 experiment sequenced for Pfs48/45
Ref Reference allele (3D7 and NF54)
Alt Alternative allele
MOI Multiplicity of infection
ND Not determined

Supplementary Table 3. Non-synonymous SNPs in *Pfs230* in sequenced oocysts

	E1152K	H1159D	H1180Y	Y1194S	Q1196E	N1209Y	Q1250K	K1254E				
Sample ID	NF54 G NF135 G NF175 A	NF54 C NF135 C NF175 G	NF54 C NF175 T NF135 T	NF54 A NF175 C NF135 C	NF54 C NF135 C NF175 G	NF54 A NF135 A NF175 T	NF54 C NF175 A NF135 A	NF54 A NF175 A NF135 G	Number of SNPs	Patient ID	mAb 2A2 condition	MOI
B03	A/G	C/G	T	C	C	A	A	G	6	BF18.002	Control	5
B42	G	C	T	C	C	A	A	G	4	BF18.003	Control	ND
B13	G	C	T	C	C	A	A	G	4	BF18.004	Control	5
B17	A	G	T	C	G	T	A	G	8	BF18.004	Control	5
B18	A	G	T	C	G	T	A	G	8	BF18.004	Control	5
B22	G	G	T	C	C	A	A	G	5	BF18.005	Control	ND
B46	G	G	T	C	C	A	A	G	5	BF18.005	Control	ND
B25	G	S	T	A/C	C	A	A	A/G	5	BF18.006	Control	ND
B26	G	C	T	A	C	A	A	A	2	BF18.006	Control	ND
B34	G	C	T	C	C	T	A	A	4	BF18.007	Control	2
B52	A/G	G	T	C	C	A	A	A	5	BF18.008	Control	5
B58	G	C	T	C	C	A	A	G	4	BF18.009	Control	ND
B60	G	C	T	C	C	A	A	G	4	BF18.009	Control	ND
B73	A/G	C/G	T	C	C	T	A	A/G	7	BF18.012	Control	6
B74	A/G	C/G	T	C	C	T	A	A/G	7	BF18.012	Control	6
B75	A/G	G	T	C	C	T	A	A	6	BF18.012	Control	6
B12	G	C	T	C	C	A	A	G	4	BF18.003	15 µg/mL	ND
B63	G	C	T	C	C/G	A/T	A/C	A/G	6	BF18.003	15 µg/mL	ND
B64	G	C	T	C	C/G	A/T	A/C	A	5	BF18.003	15 µg/mL	ND
B31	G	C	T	A	C	A	A	A	2	BF18.006	15 µg/mL	ND
B32	G	C/G	T/C	A	C	A	A	A	3	BF18.006	15 µg/mL	ND
B49	G	G	C	A	C	A	A	A	2	BF18.006	15 µg/mL	ND
B35	G	C	T	C	C	T	A	A	4	BF18.007	15 µg/mL	2
B36	G	C	T	C	C	A	A	A	3	BF18.007	15 µg/mL	2
B66	G	C	T	C	C	T	A	A	4	BF18.007	15 µg/mL	2
B67	G	C	T	C	C	T	A	A	4	BF18.007	15 µg/mL	2
B68	G	G	T	C	C	A	A	A	4	BF18.008	15 µg/mL	5
B71	A/G	G	T	C	C	A	A	A	5	BF18.008	15 µg/mL	5
B76	A/G	C/G	T	C	C	T	A	A/G	7	BF18.012	15 µg/mL	6
B77	A/G	C/G	T	C	C	T	A	A/G	7	BF18.012	15 µg/mL	6
B78	G	C/G	T	C	C	T	A	A/G	6	BF18.012	15 µg/mL	6

Occasionally, two different nucleotides are called. These are either due to the presence of single 'mixed' oocysts, derived from the gametes of two distinct clones present in the respective donor (MOI>1). These mixed signal oocysts were counted as non-synonymous SNPs. MOI: Multiplicity of infection; ND: Not Determined.

Supplementary Table 4. Oligos used for amplification and sequencing of antigen loci. Note that for sequencing of single Pfs230 domains, the gene (domain I-XIII) was PCR amplified and subsequently single domains were sequenced with indicated primers.

	**5'-3' sequence**	**PCR**	**Domain amplified**	**Used for sequencing**	**Domain sequenced**
Pfs25 Oocysts	TTATTCTTTTAAAAATGAATAAACTTTAC	Nested 1 forward	Full length		
	ATTTACATTATAAAAAAGCATACTGA	Nested 1 reverse	Full length		
	ATGAATAAACTTTACAGTTTGTTTCT	Nested 2 forward	Full length	Yes	Full length
	TGAAAATAGTATAAACATAATGCTTAG	Nested 2 reverse	Full length		
Pfs48/45 Oocysts	ATGTACGTGTATTAAAATATCC	Nested 1 forward	6C		
	GATCTTTTTACATATTTGCCG	Nested 2 forward	6C	Yes	6C
	TATAATAATATTGCTACAATTAGG	Nested 1&2 reverse	6C		
Pfs230 Oocysts	CATTTCAGGTTCCACCATATATAG	Nested 1 forward	IV		
	GATCTTTGGCTGGACAAAATATTTG	Nested 1 reverse	IV		
	ACCTTTTTATTTTATGTTTGGTTG	Nested 2 forward	IV	Yes	IV
	ATCAAATGTTTTATTTACTCTTGG	Nested 2 reverse	IV		
Pfs230 Parasite strains	GGAGATACTGCTGTATCCGAAG	Forward	I – XIV	Yes	II
	GATGGCTCTTGATTGAGGTTCTGG	Reverse	I – XIV	Yes	XIII
	GATTGTAGAAGTGTATGTAGAACC			Yes	II (forward)
	GAATACCACCTATATCTCCGCTA			Yes	II (reverse)
	CATTTCAGGTTCCACCATATATAG			Yes	IV (forward)
	GATCTTTGGCTGGACAAAATATTTG			Yes	IV (reverse)
	AGCTCCAAAATTAATGATGTCTGC			Yes	VII (forward)
	CATTATCACATATGATACCAAATACT			Yes	VII (reverse)
	CACTACACTACCAACTGATGGTG			Yes	XII (forward)
	GGCTCCTGGTAAGATATGTTCTA			Yes	XII (reverse)
	GTTGCTACTTGTGAAATTATTGATAC			Yes	XIII (forward)

Supplementary Table 5. Regression models fitted to membrane feeding data. For each antigen, our starting point was a simple model, using the same mean value of the slope for all the data (parameter a). This parameter contains a random effect for the experiment (SMFA data) or the donor (DMFA). We then fitted more complicated models, allowing the slope to vary depending on the assay used and, in the case of the DMFA data, the country in which the experiment was carried out. We then assessed the goodness of fit using the widely applicable information criterion (WAIC), showing the disparity (ΔWAIC) in the fit between each model and the best-performing model. The corresponding Akaike weights, indicating the weight that each model is attributed in the ensemble of models used to generate Figure 1. We here summarise the slopes obtained from the model ensemble. For mAb 4B7, the slope for the SMFA model was 0.126, and for the DMFA model 0.124 (both countries). For mAb 45.1, the slope for the SMFA model was 0.521, for the DMFA models it was 0.476 (BF) and 0.538 (CAM). For mAb 2A2, the slope is as stated in the table (0.510), as we did not include the DMFA data in the regression modelling carried out here, because only two datapoints per donor were available. SMFA: Standard Membrane Feeding assay; DMFA: Direct Membrane Feeding Assay; BF: Burkina Faso; CAM: Cameroon.

Model [parameters]	ΔWAIC	Akaike Weight	Parameter values (95% CI)
**4B7 (Pfs25)**			
One slope for all data [a]	-	0.37	a=0.12 (0.11, 0.14)
Slope correction for the assay ([b]: SMFA=0, DMFA=1)	1.0	0.23	a=0.13 (0.08, 0.17),<br>b=-0.01 (-0.05, 0.04)
Slope correction for the country for the DMFA data ([d]: BF=0, CAM=1)	1.1	0.21	a=0.13 (0.08, 0.18),<br>d=0.0 (-0.03, 0.03)
Slope correction for the assay ([b]: SMFA=0, DMFA=1), and the country for the DMFA data ([d]: BF=0, CAM=1)	1.3	0.19	a=0.13 (0.08, 0.18),<br>b=-0.01 (-0.06, 0.05),<br>d=0.00 (-0.03, 0.04)
**45.1 (Pfs48/45)**			
Slope correction for the assay ([b]: SMFA=0, DMFA=1), and the country for the DMFA data ([d]: BF=0, CAM=1)	-	0.43	a=0.54 (0.48, 0.59),<br>b=-0.09 (-0.15, -0.01),<br>d=0.10 (0.03, 0.16)
Slope correction for the country of origin for the DMFA data ([d]: BF=0, CAM=1)	0.8	0.29	a=0.49 (0.45, 0.53),<br>d=0.06 (0.0, 0.12)
One slope for all data [a]	2.0	0.16	a=0.51 (0.47, 0.54)
Slope correction for the assay ([b]: SMFA=0, DMFA=1)	2.6	0.12	a=0.54 (0.46, 0.61),<br>b=-0.04 (-0.12, 0.05)
**2A2 (Pfs230)**			
One slope for all data [a]	-	1	a=0.51 (0.38, 0.64)

Supplementary Table 6. Slopes of the regression models fitted to the standard membrane feeding data of monoclonal antibody 2A2 with lab-adapted parasite strains. For NF175 and NF176, the 95% credible intervals (CI) of the slope include zero, because of this the 80% inhibitory concentration presented in Figure 3 is an estimate and no credible intervals are given.

**Strain**	**Slope (95% CI)**
NF54	0.51 (0.38, 0.64)
NF135	0.31 (0.22, 0.40)
NF183	0.35 (0.26, 0.45)
NF175	0.01 (0, 0.02)
NF176	0.03 (0, 0.05)
NF149	0.05 (0.02, 0.09)

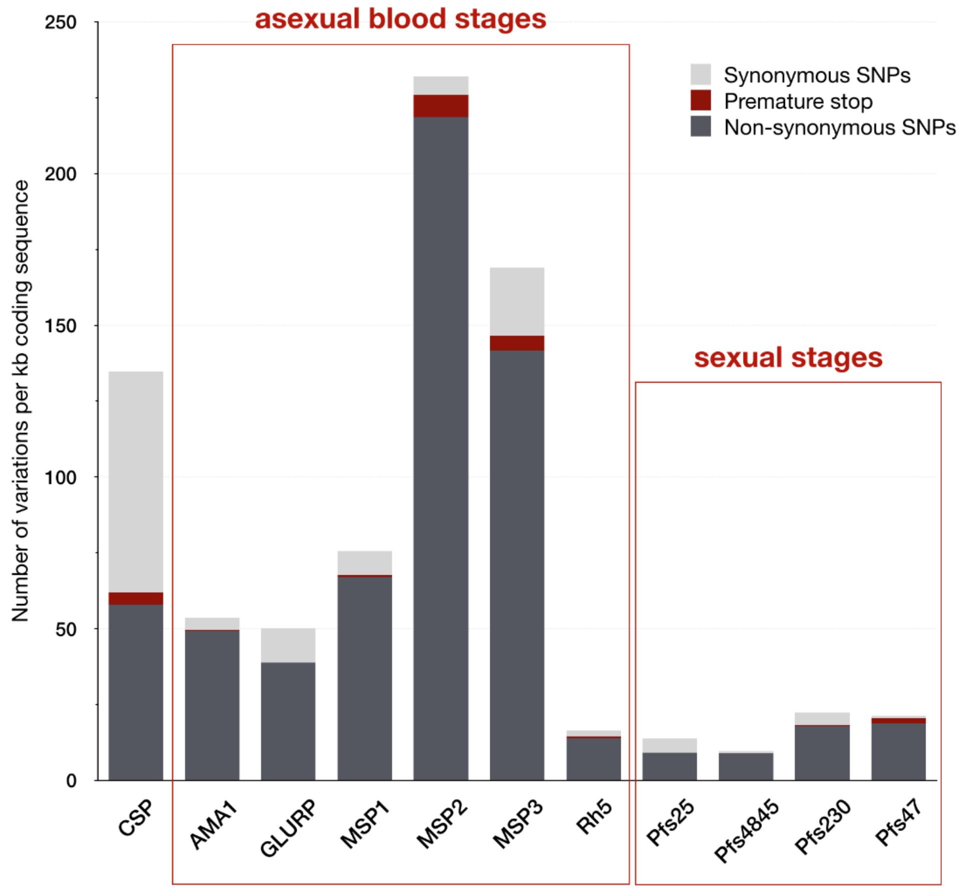

Supplementary Figure 1. Genetic variation of *Plasmodium falciparum* vaccine candidates and targets. Pre-erythrocytic stage (CSP), asexual blood stage and sexual stage targets are depicted. Non-synonymous, synonymous Single Nucleotide Polymorphisms (SNPs) and pre-mature stops (nonsense-mutations) as reported on PlasmoDB (v.46) are indicated in dark grey, light grey and red, respectively. All SNP counts were normalized to the length of the coding sequence.

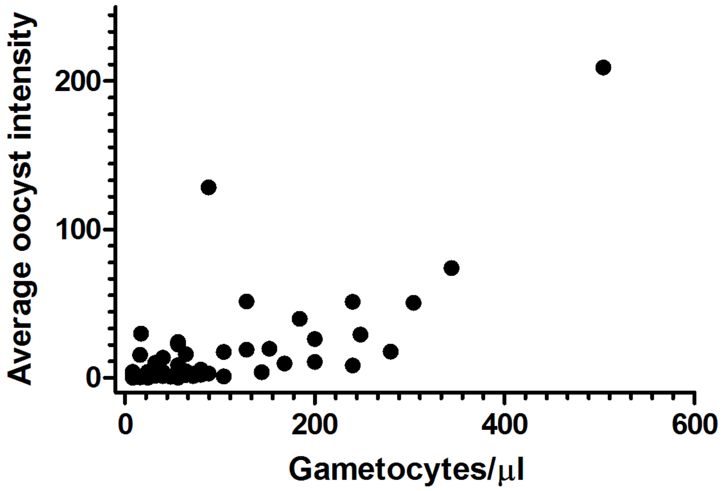

Supplementary Figure 2. Gametocyte density in relation to oocyst intensity in DMFA experiments. Correlation (Spearman's rank correlation coefficient = 0.67, p<0.0001) of gametocyte densities determined by light microscopy are plotted against the average oocyst intensity (oocysts/mosquito) as observed in direct membrane feeding assays (DMFA). DMFAs were performed with blood in which autologous plasma was replaced by naïve serum (=antibody control).

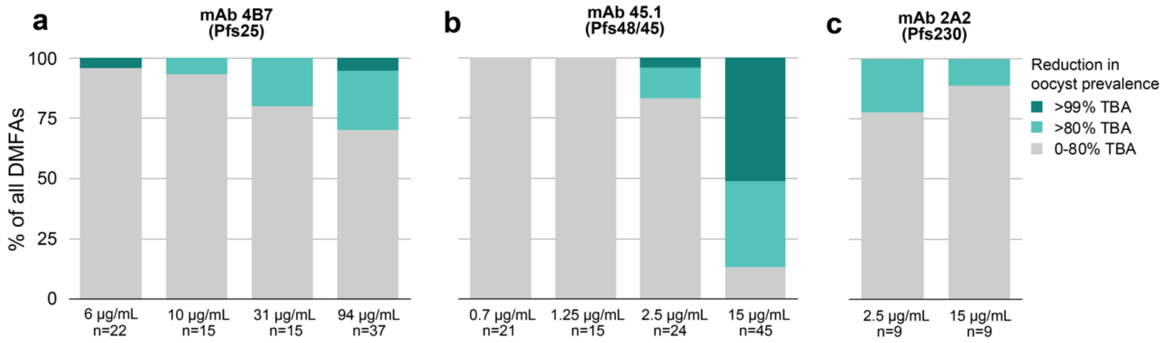

Supplementary Figure 3. Transmission-blocking activity of three different monoclonal antibodies in direct membrane feeding assay on naturally infected gametocyte carriers. The concentration of *(a)* monoclonal antibody (mAb) 4B7 (Pfs25), *(b)* mAb 45.1 (Pfs48/45), *(c)* mAb 2A2 (Pfs230) in the feeder is indicated. Transmission-blocking activity was quantified as a reduction in the percentage of mosquitoes with oocysts present between antibody control and mAb condition for each donor. Complete transmission-blocking (>99 % TBA) is depicted in dark petrol and moderate reduction (80-99% TBA) in cyan. All donors with >1 oocyst/mosquito (i.e. successful transmission) and >30% mosquito prevalence in antibody control condition were included from two sites (Burkina Faso and Cameroon), except for mAb 2A2 *(c)* which was only tested in Burkina Faso. The number of distinct donor samples (n) tested for each antibody is given below bars. DMFA: Direct Membrane Feeding Assay.

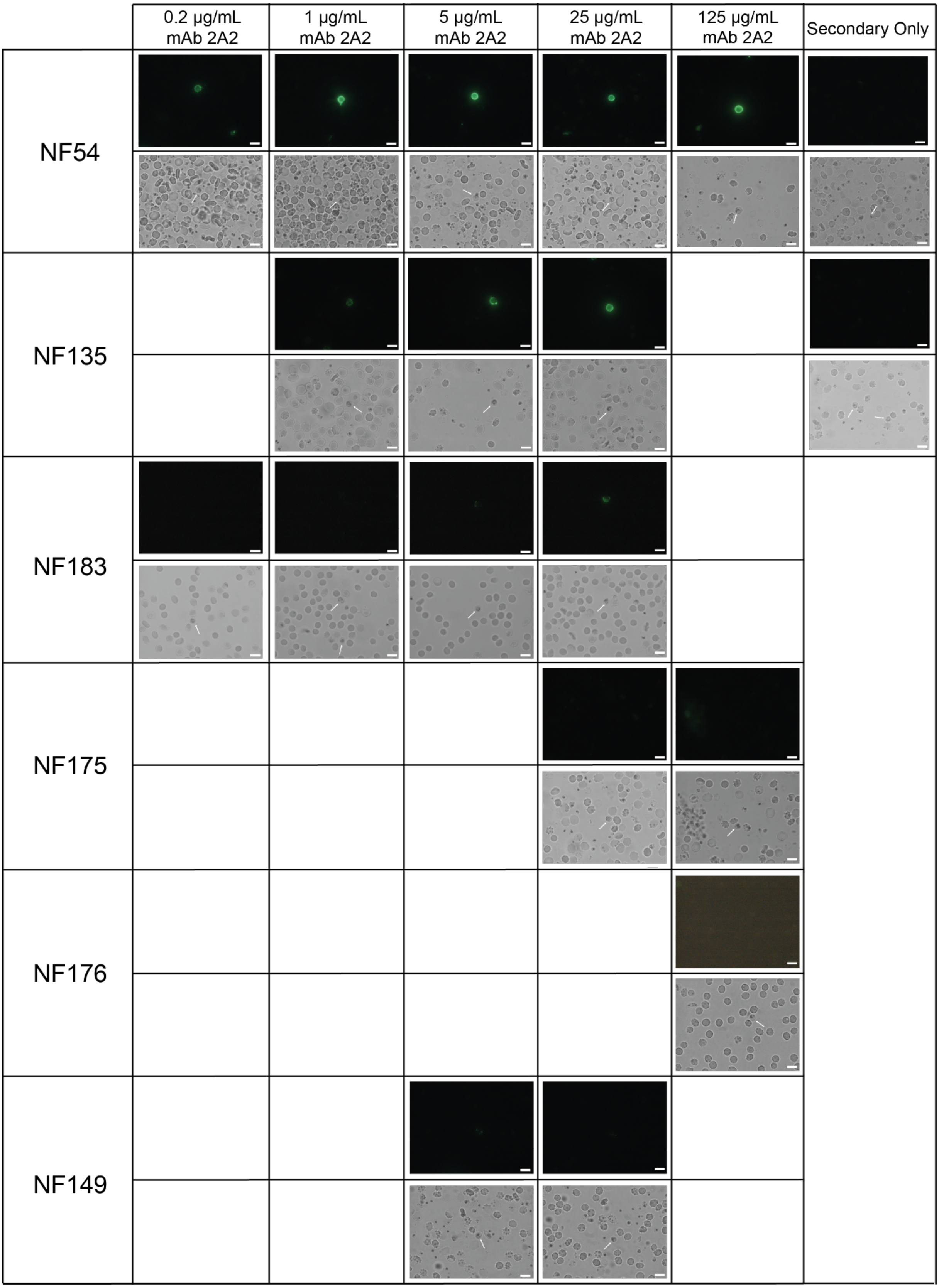

Supplementary Figure 4. Antibody binding of monoclonal antibody (mAb) 2A2 to the female gamete surface. Six lab-adapted parasite strains were incubated with different concentrations of Pfs230 mAb 2A2 and imaged using fluorescent microscopy. White arrows in bright-field images indicate activated female gametes. Scale bar represent 20µm.

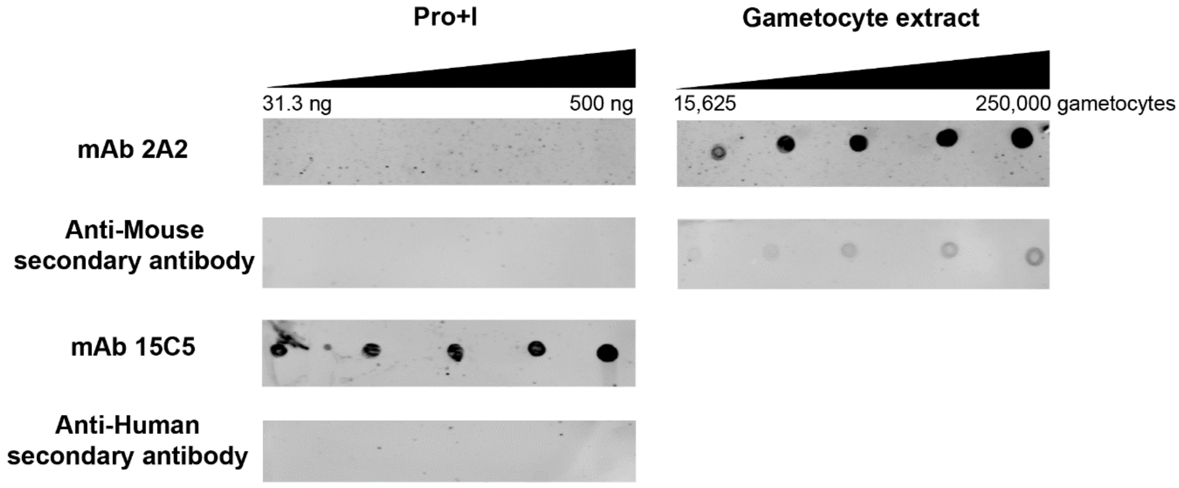

Supplementary Figure 5. Dot blot of mAb 2A2 with recombinant Pfs230 Pro+I and gametocyte extract.

Serial dilutions of recombinant protein 230CMB (Pro+I, aa444-730) (1) and gametocyte extract were spotted on nitrocellulose membranes. After drying, the membranes were blocked for one hour with 5% milk in PBS. Subsequently, incubated for one hour with 5 µg/mL mAb 2A2 or mAb 15C5 in 1% milk in PBS with 0.1% Tween20. The blots were washed three times with PBS with 0.1% Tween20 and incubated for one hour with 1:10,000 Goat Anti-Mouse IRDye680RD (for mAb 2A2) (LI-COR, Cat. No. 926-68070) or Goat Anti-Human IRDye680 (for mAb 15C5) (LI-COR, Cat. No. 926-68078). The membranes were washed 3 times with PBS with 0.1% Tween20 and ones with PBS before imaging on the Odyssey CLx (Li-COR). mAb 15C5 is a conformational humanized sister mAb of the published mAb 15A4 and was a kind gift of MVI-Path (2).

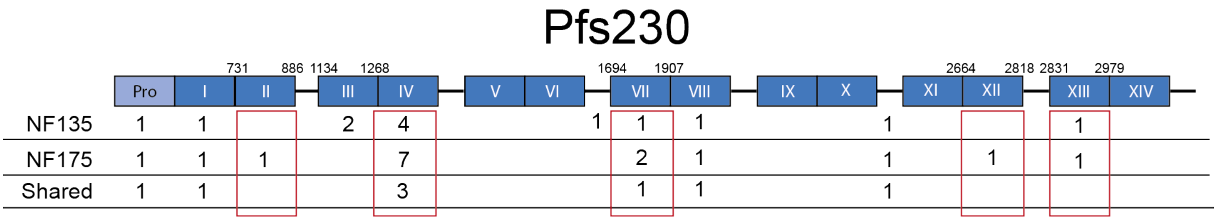

Supplementary Figure 6. Schematic representation of Pfs230. Domains are indicated with roman numbers. Number of non-synonymous Single Nucleotide Polymorphisms (SNPs), compared to the reference strains NF54, in each domain are shown for NF135 and NF175. SNPs that are shared by both strains are shown in the bottom row. Based on this analysis, domains highlighted with a red box were selected for sanger sequencing of other lab-adapted parasite strains (NF149, NF183 and NF176). Amino acid numbers for these domains were retrieved from Gerloff et al. (3).

Domain II (amino acids 731 – 886)

```
NF54       GNKINGCAFLDEDEEEEKYGNQIEEDEHNEKIKMKTFFTQNIYKKNNIYPCYMKLYSGDI      60
NF135      ............................................................      60
NF183      ............................................................      60
NF175      .................I..........................................      60
NF176      ............................................................      60
NF149      ............................................................      60

NF54       GGILFPKNIKSTTCFEEMIPYNKEIKWNKENKSLGNLVNNSVVYNKEMNAKYFNVQYVHI      120
NF135      ............................................................      120
NF183      ............................................................      120
NF175      ............................................................      120
NF176      ............................................................      120
NF149      ................K...........................................      120

NF54       PTSYKDTLNLFCSIILKEEESNLISTSYLVYVSINE 156
NF135      .................................... 156
NF183      .................................... 156
NF175      .................................... 156
NF176      .................................... 156
NF149      .................................... 156
```

Domain VII (amino acids 1694-1907)

```
NF54       NRHVCDFSKNNLIVPESLKKKEELGGNPVNIHCYALLKPLDTLYVKCPTSKDNYEAAKVN     60
NF135      ............................................................      60
NF183      ............................................................      60
NF175      ............................................................      60
NF176      ............................................................      60
NF149      ............................................................      60

NF54       ISENDNEYELQVISLIEKRFHNFETLESKKPGNGDVVVHNGVVDTGPVLDNSTFEKYFKN      120
NF135      ............................................................      120
NF183      ..........................................L.................      120
NF175      ............................................................      120
NF176      ............................................................      120
NF149      ............................................................      120

NF54       IKIKPDKFFEKVINEYDDTEEEKDLESILPGAIVSPMKVLKKKDPFTSYAAFVVPPIVPK      180
NF135      ...............N............................................      180
NF183      ...............N........................................V...      180
NF175      ...............N........................................V...      180
NF176      ...............N............................................      180
NF149      ...............N............................................      180

NF54       DLHFKVECNNTEYKDENQYISGYNGIIHIDISNS   214
NF135      ..................................   214
NF183      ..................................   214
NF175      ..................................   214
NF176      ..................................   214
NF149      ..................................   214
```

Domain XII (amino acids 2664-2818)

```
NF54       RNIIHGCDFLYLENQTNDAISNNNNNSYSIFTHNKNTENNLICDISLIPKTVIGIKCPNK      60
NF135      ............................................................      60
NF183      ............................................................      60
NF175      ............................................................      60
NF176      ............................................................      60
NF149      ............................................................      60

NF54       KLNPQTCFDEVYYVKQEDVPSKTITADKYNTFSKDKIGNILKNAISINNPDEKDNTYTYL      120
NF135      ............................................................      120
NF183      ............................................................      120
NF175      ...................................................D........      120
NF176      ............................................................      120
NF149      ............................................................      120

NF54       ILPEKFEEELIDTKKVLACTCDNKYIIHMKIEKST  155
NF135      ...................................  155
NF183      ...................................  155
NF175      ...................................  155
NF176      ...................................  155
NF149      ...................................  155
```

Domain XIII (amino acids 2831-2979)

```
NF54     GKDICKYDVTTKVATCEIIDTIDSSVLKEHHTVHYSITLSRWDKLIIKYPTNEKTHFENF     60
NF135    .......................................L....................     60
NF183    .......................................L....................     60
NF175    ............................................................     60
NF176    ............................................................     60
NF149    .......................................L....................     60

NF54     FVNPFNLKDKVLYNYNKPINIEHILPGAITTDIYDTRTKIKQYILRIPPYVHKDIHFSLE     120
NF135    ............................................................     120
NF183    ............................................................     120
NF175    ............................................................     120
NF176    ............................................................     120
NF149    ............................................................     120

NF54     FNNSLSLTKQNQNIIYGNVAKIFIHINQG       149
NF135    .............................       149
NF183    .............................       149
NF175    .............N...............       149
NF176    .............................       149
NF149    .............................       149
```

Supplementary Figure 7. Amino acid sequence alignments of six lab-cultured parasite strains for Pfs230 domains. Amino acid residues that differ from NF54 are specified. Domain boundaries were retrieved from Gerloff et al. (3). Identical amino acids are indicated by dots.

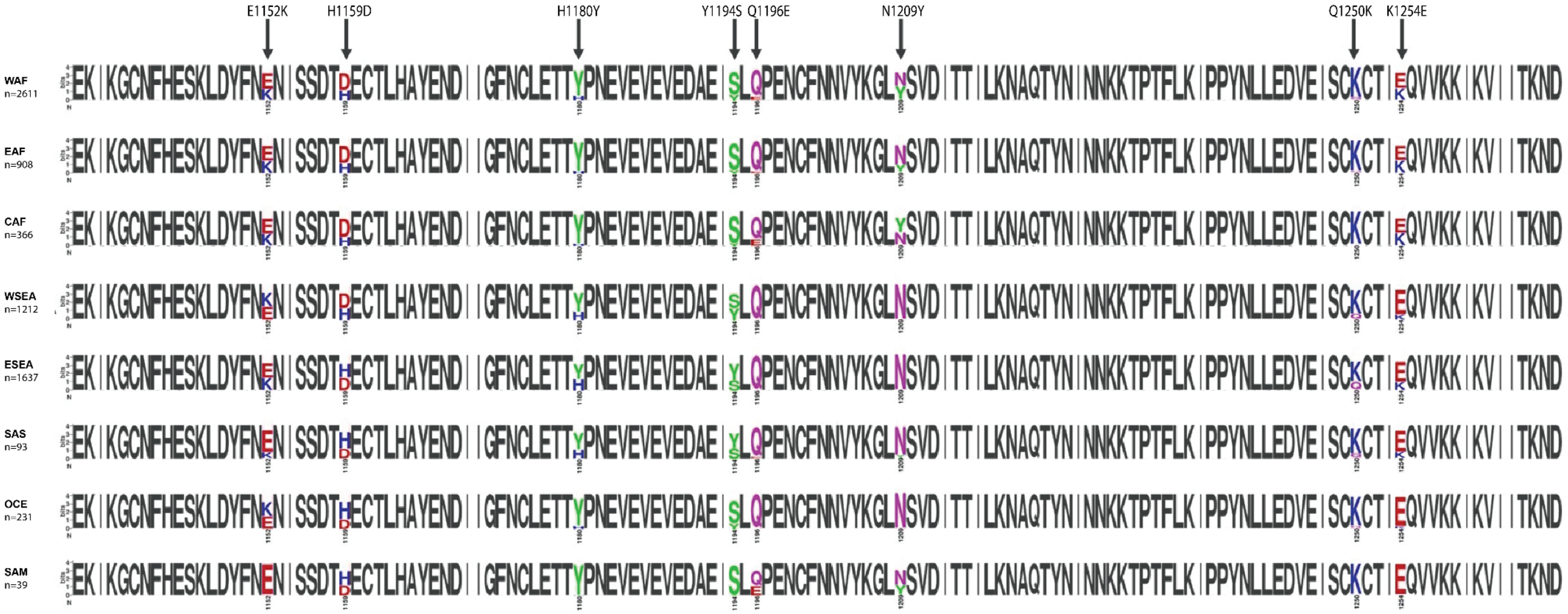

Supplementary Figure 8. Sequence variation Pfs230 domain IV per geographical region. Variation in amino acid sequence of domain IV of Pfs230 in the regions covered by the malariagen Pf3k project (https://www.malariagen.net/apps/pf3k/release_3/index.html). WAF: West Africa, EAF: East Africa, CAF: Central Africa, WSEA: Western South East Asia, ESEA: Eastern South East Asia, SAS: South Asia, OCE: Oceania, SAM: South America. All but eight residues (indicated by arrows and coloured amino acid code) are highly conserved.

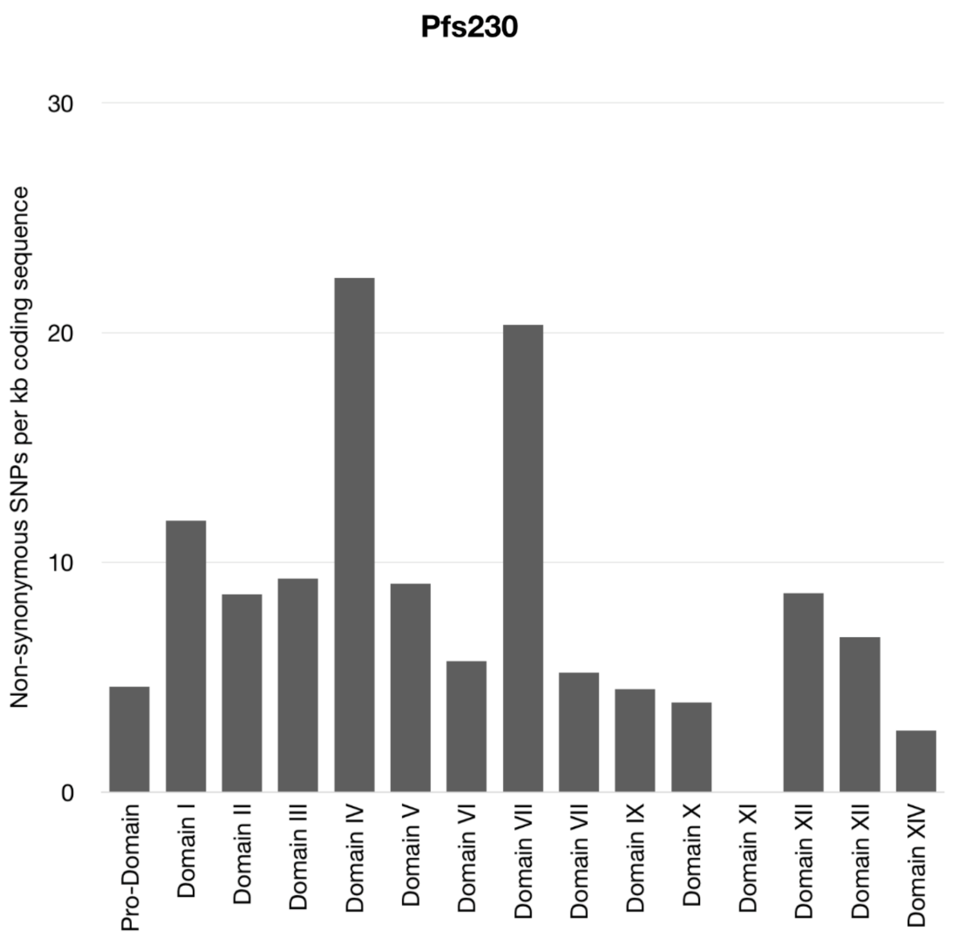

Supplementary Figure 9. Genetic variation in the protein domains of Pfs230. Domain boundaries were retrieved from Gerloff et al. (3). Non-synonymous Single Nucleotide Polymorphisms (SNPs) as reported by PlasmoDB v46 were counted and normalized for the length of the respective coding sequence.

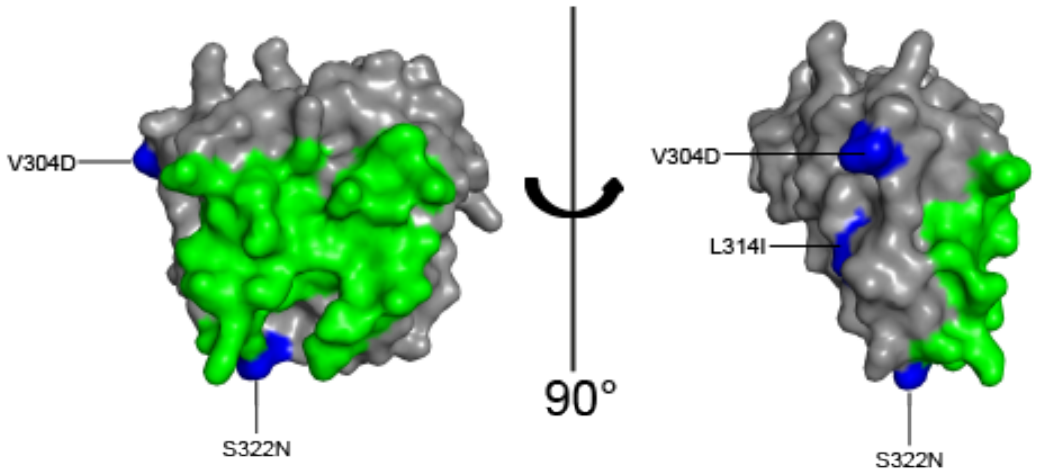

Supplementary Figure 10. Crystal structure surface presentations of the 6C domain of Pfs48/45 (PDB id: 6E62). Amino acid mutations that were identified by oocyst sequencing are highlighted in blue. The antibody binding sites are shown in green and are based on the crystal structure of a Pfs48/45-6C complex with Fab 45.1 (4).

Supplementary References

1. Farrance CE, Rhee A, Jones RM, Musiychuk K, Shamloul M, Sharma S, et al. A plant-produced Pfs230 vaccine candidate blocks transmission of Plasmodium falciparum. Clin Vaccine Immunol. 2011;18(8):1351-7.
2. Lee SM, Plieskatt J, Krishnan S, Raina M, Harishchandra R, King CR. Expression and purification optimization of an N-terminal Pfs230 transmission-blocking vaccine candidate. Protein Expr Purif. 2019;160:56-65.
3. Gerloff DL, Creasey A, Maslau S, Carter R. Structural models for the protein family characterized by gamete surface protein Pfs230 of Plasmodium falciparum. Proc Natl Acad Sci U S A. 2005;102(38):13598-603.
4. Kundu P, Semesi A, Jore MM, Morin MJ, Price VL, Liang A, et al. Structural delineation of potent transmission-blocking epitope I on malaria antigen Pfs48/45. Nat Commun. 2018;9(1):4458.
